# Usefulness of inserting a modified single‐pigtail plastic stent into a metallic stent in endoscopic ultrasound-guided gallbladder drainage

**DOI:** 10.1055/a-2163-1828

**Published:** 2023-09-27

**Authors:** Koichi Soga

**Affiliations:** 1 Dokkyo Medical University Saitama Medical Center, Department of Gastroenterology, Saitama, Japan; 2 Omihachiman Community Medical Center, Department of Gastroenterology, Shiga, Japan

Endoscopic ultrasound-guided gallbladder drainage (EUS-GBD) has emerged as an alternative drainage technique, especially for high-risk surgical patients 1 . Sequential insertion of the stent delivery system into the gallbladder is a crucial technical step for successful EUS-GBD 2 3 .

EUS-GBD was performed on a 67-year-old Japanese man who presented with cholecystitis and concurrent biliary tract cancer during chemotherapy. The gallbladder was punctured from the duodenum using a 19G fine needle. The puncture tract was dilated using an electrocautery dilator, and a fully covered self-expandable metal stent (FCSEMS; Bonastent M-intraductal biliary stent, 8 mm × 6 cm; Standard Sci-Tech, Korea) was deployed ( Fig. 1 ). To prevent food impaction and gallbladder wall injury by the FCSEMS 1 , insertion of a 7‐Fr double‐pigtail plastic stent (DPPS; Through & Pass Stent, Gadelius Medical K.K., Japan) into the FCSEMS was attempted. However, the DPPS caught at the edge of the FCSEMS at the duodenal side and again at the sclerotic gallbladder after passing through; thus, DPPS insertion was abandoned. The DPPS had a step between the inner sheath and the DPPS itself, and the DPPS tip bend was stiff, suggesting that the insertion axis of the DPPS could change. Therefore, insertion of a modified single-pigtail plastic stent (mSPPS) was attempted instead. The mSPPS was improvised from a 6-Fr endoscopic nasobiliary drainage tube (Flexima, Boston Scientific, USA), shortening it to 15 cm from the straight section. Compared with the DPPS, the mSPPS had a narrower tip at the top that gradually widened; this improved insertion by reducing the chance of the tube getting stuck in the FCSEMS and sclerotic gallbladder 3 ( Fig. 2 , Video 1 ). No procedural complications occurred, and the patient’s clinical course was good. This case demonstrates the usefulness of a novel self-made mSPPS inserted into a FCSEMS in the EUS-GBD procedure.

**Fig. 1:**
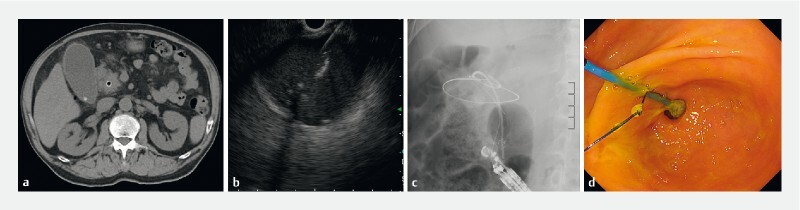
Usefulness of modified single‐pigtail plastic stent inserted into a fully covered self-expandable metal stent (FCSEMS) in endoscopic ultrasound-guided gallbladder drainage. **a** Abdominal computed tomography (CT) shows an enlarged gallbladder, increased CT values in the surrounding fatty tissue, and gallbladder stones originating from acute cholecystitis. **b** The gallbladder is punctured from the duodenal bulb using a 19G fine needle under endoscopic ultrasound guidance. **c** A modified single-pigtail plastic stent (mSPPS), instead of a double-pigtail plastic stent, is deployed within the FCSEMS. **d** The endoscopic image shows that the mSPPS is correctly implanted within the FCSEMS without any complications.

**Fig. 2:**
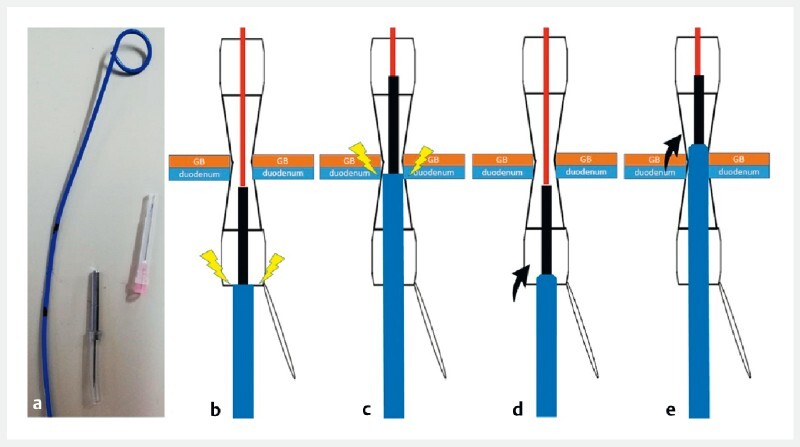
Concept of the mSPPS. **a** The mSPPS is improvised from a commercial 6-Fr endoscopic nasobiliary drainage tube. **b, c** The double-pigtail plastic stent (DPPS) catches at the edge of the FCSEMS at the duodenal side ( **b** ) and again, after passing through, at the sclerotic gallbladder and duodenal puncture site ( **c** ). **d, e** The mSPPS made from an endoscopic nasobiliary drainage tube is narrower at the tip than the DPPS, improving insertion by limiting the chance of the tube getting stuck in the edge of the FCSEMS at the duodenal side ( **d** ) and at the sclerotic gallbladder and duodenal puncture site ( **e** ).

**Video 1** Endoscopic ultrasound-guided gallbladder drainage procedure with a modified single-pigtail plastic stent (mSPPS) inserted into a fully covered self-expandable metal stent. The mSPPS is improvised from a commercial 6-Fr endoscopic nasobiliary drainage tube that was shortened to 15 cm from the straight section. For mSPPS insertion, the remaining endoscopic nasobiliary drainage tube and a guidewire are used as a pusher catheter.


Endoscopy_UCTN_Code_TTT_1AS_2AD

## Notes

Endoscopy E-Videos

https://eref.thieme.de/e-videos
*E-Videos* is an open access online section of the journal *Endoscopy* , reporting on interesting cases and new techniques in gastroenterological endoscopy. All papers include a high-quality video and are published with a Creative Commons CC-BY license. Endoscopy E-Videos qualify for HINARI discounts and waivers and eligibility is automatically checked during the submission process. We grant 100% waivers to articles whose corresponding authors are based in Group A countries and 50% waivers to those who are based in Group B countries as classified by Research4Life (see: https://www.research4life.org/access/eligibility/ ). This section has its own submission website at https://mc.manuscriptcentral.com/e-videos
